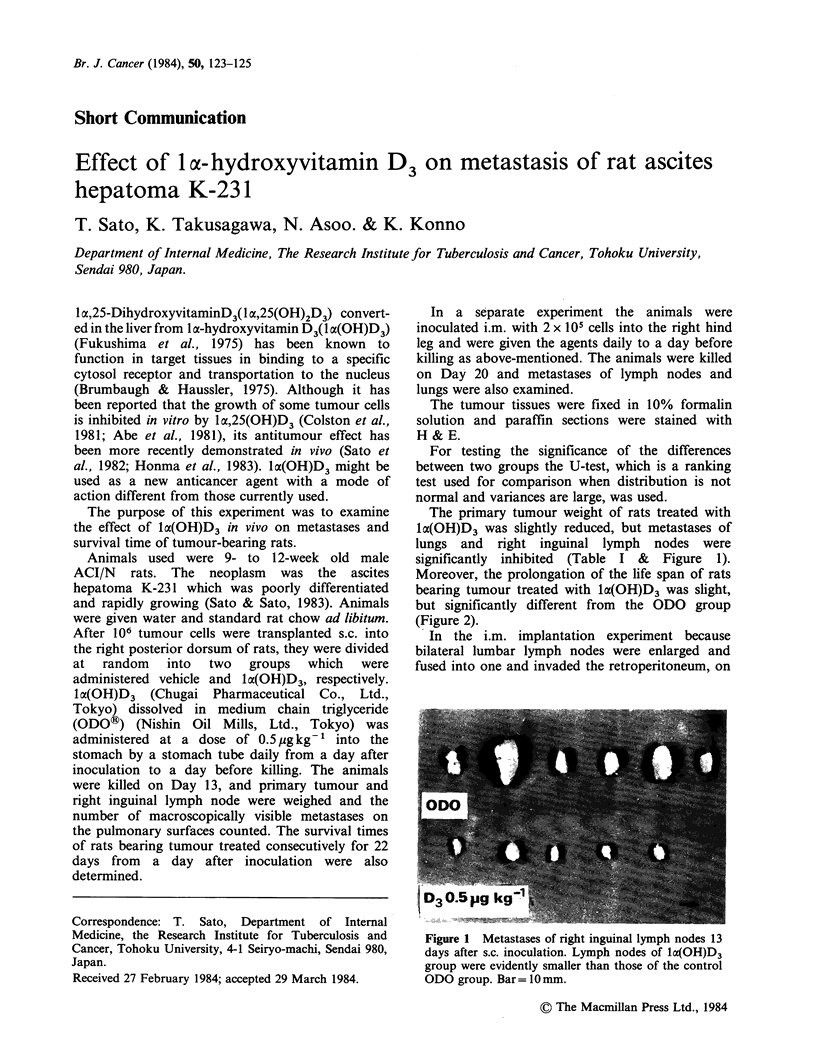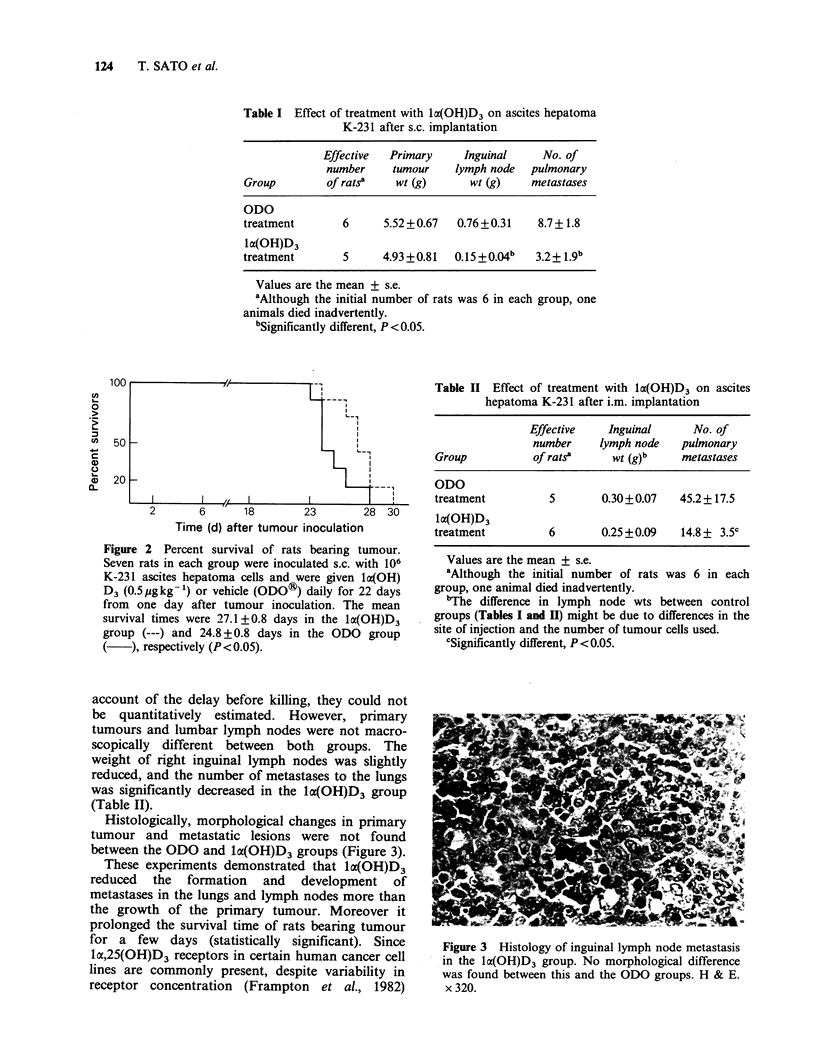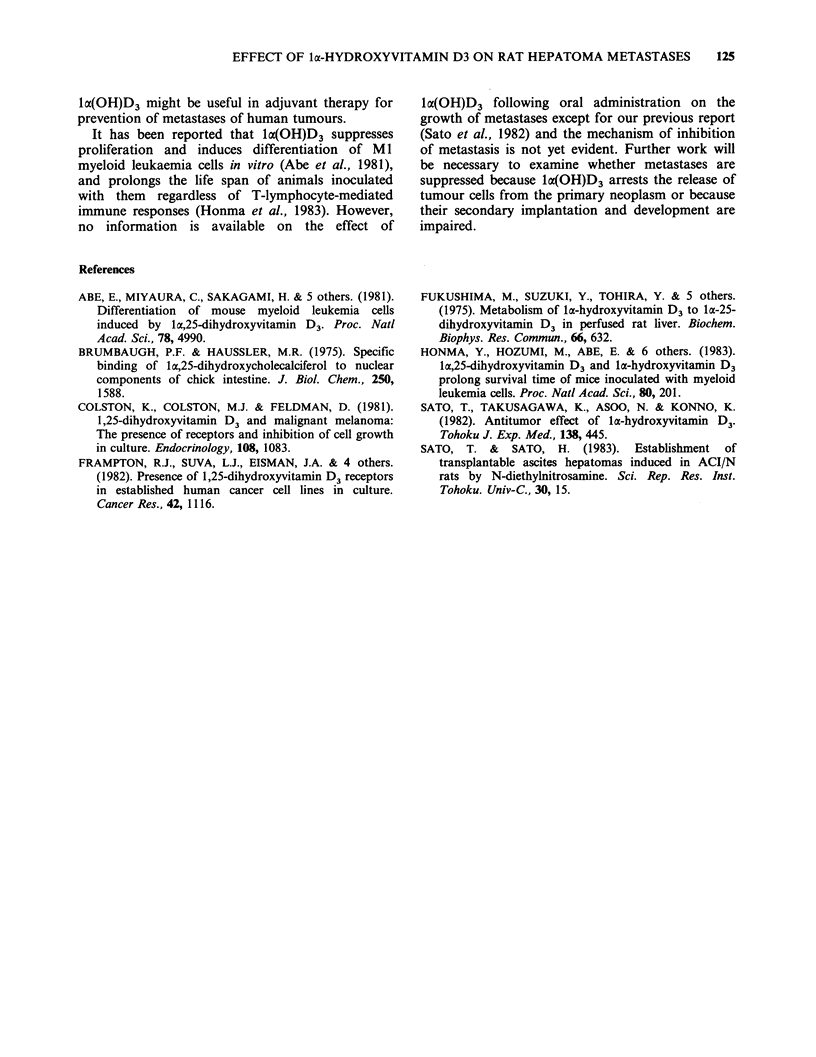# Effect of 1 alpha-hydroxyvitamin D3 on metastasis of rat ascites hepatoma K-231.

**DOI:** 10.1038/bjc.1984.148

**Published:** 1984-07

**Authors:** T. Sato, K. Takusagawa, N. Asoo, K. Konno

## Abstract

**Images:**


*Br. J. Cancer* (1984), **50**, 123–125

### Short Communication

### Effect of 1α-hydroxyvitamin $D_3$ on metastasis of rat ascites hepatoma K-231

T. Sato, K. Takusagawa, N. Asoo. & K. Konno

*Department of Internal Medicine, The Research Institute for Tuberculosis and Cancer, Tohoku University, Sendai 980, Japan.*

1α,25-Dihydroxyvitamin$D_3$(1α,25$(OH)_2D_3$) converted in the liver from 1α-hydroxyvitamin $D_3$(1α$(OH)D_3$) (Fukushima *et al.,* 1975) has been known to function in target tissues in binding to a specific cytosol receptor and transportation to the nucleus (Brumbaugh & Haussler, 1975). Although it has been reported that the growth of some tumour cells is inhibited *in vitro* by 1α,25$(OH)D_3$ (Colston *et al.,* 1981; Abe *et al.,* 1981), its antitumour effect has been more recently demonstrated *in vivo* (Sato *et al.,* 1982; Honma *et al.,* 1983). 1α$(OH)D_3$ might be used as a new anticancer agent with a mode of action different from those currently used.

The purpose of this experiment was to examine the effect of 1α$(OH)D_3$ *in vivo* on metastases and survival time of tumour-bearing rats.

Animals used were 9- to 12-week old male ACI/N rats. The neoplasm was the ascites hepatoma K-231 which was poorly differentiated and rapidly growing (Sato & Sato, 1983). Animals were given water and standard rat chow *ad libitum.* After $10^6$ tumour cells were transplanted s.c. into the right posterior dorsum of rats, they were divided at random into two groups which were administered vehicle and 1α$(OH)D_3$, respectively. 1α$(OH)D_3$ (Chugai Pharmaceutical Co., Ltd., Tokyo) dissolved in medium chain triglyceride (ODO®) (Nishin Oil Mills, Ltd., Tokyo) was administered at a dose of $0.5\,\mu g\,kg^{-1}$ into the stomach by a stomach tube daily from a day after inoculation to a day before killing. The animals were killed on Day 13, and primary tumour and right inguinal lymph node were weighed and the number of macroscopically visible metastases on the pulmonary surfaces counted. The survival times of rats bearing tumour treated consecutively for 22 days from a day after inoculation were also determined.

Correspondence: T. Sato, Department of Internal Medicine, the Research Institute for Tuberculosis and Cancer, Tohoku University, 4-1 Seiryo-machi, Sendai 980, Japan.

Received 27 February 1984; accepted 29 March 1984.

In a separate experiment the animals were inoculated i.m. with $2\times10^5$ cells into the right hind leg and were given the agents daily to a day before killing as above-mentioned. The animals were killed on Day 20 and metastases of lymph nodes and lungs were also examined.

The tumour tissues were fixed in 10% formalin solution and paraffin sections were stained with H & E.

For testing the significance of the differences between two groups the U-test, which is a ranking test used for comparison when distribution is not normal and variances are large, was used.

The primary tumour weight of rats treated with 1α$(OH)D_3$ was slightly reduced, but metastases of lungs and right inguinal lymph nodes were significantly inhibited (Table I & Figure 1). Moreover, the prolongation of the life span of rats bearing tumour treated with 1α$(OH)D_3$ was slight, but significantly different from the ODO group (Figure 2).

In the i.m. implantation experiment because bilateral lumbar lymph nodes were enlarged and fused into one and invaded the retroperitoneum, on

**Figure 1** Metastases of right inguinal lymph nodes 13 days after s.c. inoculation. Lymph nodes of 1α$(OH)D_3$ group were evidently smaller than those of the control ODO group. Bar = 10 mm.

© The Macmillan Press Ltd., 1984

**Table I** Effect of treatment with 1α(OH)$D_3$ on ascites hepatoma K-231 after s.c. implantation

| *Group* | *Effective number of rats*[a] | *Primary tumour wt (g)* | *Inguinal lymph node wt (g)* | *No. of pulmonary metastases* |
|---|---|---|---|---|
| ODO treatment | 6 | 5.52±0.67 | 0.76±0.31 | 8.7±1.8 |
| 1α(OH)$D_3$ treatment | 5 | 4.93±0.81 | 0.15±0.04[b] | 3.2±1.9[b] |

Values are the mean ± s.e.
[a]Although the initial number of rats was 6 in each group, one animals died inadvertently.
[b]Significantly different, $P<0.05$.

**Figure 2** Percent survival of rats bearing tumour. Seven rats in each group were inoculated s.c. with $10^6$ K-231 ascites hepatoma cells and were given 1α(OH) $D_3$ (0.5 $\mu$g kg$^{-1}$) or vehicle (ODO®) daily for 22 days from one day after tumour inoculation. The mean survival times were 27.1±0.8 days in the 1α(OH)$D_3$ group (---) and 24.8±0.8 days in the ODO group (——), respectively ($P<0.05$).

**Table II** Effect of treatment with 1α(OH)$D_3$ on ascites hepatoma K-231 after i.m. implantation

| *Group* | *Effective number of rats*[a] | *Inguinal lymph node wt (g)*[b] | *No. of pulmonary metastases* |
|---|---|---|---|
| ODO treatment | 5 | 0.30±0.07 | 45.2±17.5 |
| 1α(OH)$D_3$ treatment | 6 | 0.25±0.09 | 14.8± 3.5[c] |

Values are the mean ± s.e.
[a]Although the initial number of rats was 6 in each group, one animal died inadvertently.
[b]The difference in lymph node wts between control groups (**Tables I and II**) might be due to differences in the site of injection and the number of tumour cells used.
[c]Significantly different, $P<0.05$.

account of the delay before killing, they could not be quantitatively estimated. However, primary tumours and lumbar lymph nodes were not macroscopically different between both groups. The weight of right inguinal lymph nodes was slightly reduced, and the number of metastases to the lungs was significantly decreased in the 1α(OH)$D_3$ group (Table II).

Histologically, morphological changes in primary tumour and metastatic lesions were not found between the ODO and 1α(OH)$D_3$ groups (Figure 3).

These experiments demonstrated that 1α(OH)$D_3$ reduced the formation and development of metastases in the lungs and lymph nodes more than the growth of the primary tumour. Moreover it prolonged the survival time of rats bearing tumour for a few days (statistically significant). Since 1α,25(OH)$D_3$ receptors in certain human cancer cell lines are commonly present, despite variability in receptor concentration (Frampton et al., 1982)

**Figure 3** Histology of inguinal lymph node metastasis in the 1α(OH)$D_3$ group. No morphological difference was found between this and the ODO groups. H & E. ×320.

1α(OH)$D_3$ might be useful in adjuvant therapy for prevention of metastases of human tumours.

It has been reported that 1α(OH)$D_3$ suppresses proliferation and induces differentiation of M1 myeloid leukaemia cells *in vitro* (Abe *et al.*, 1981), and prolongs the life span of animals inoculated with them regardless of T-lymphocyte-mediated immune responses (Honma *et al.*, 1983). However, no information is available on the effect of 1α(OH)$D_3$ following oral administration on the growth of metastases except for our previous report (Sato *et al.*, 1982) and the mechanism of inhibition of metastasis is not yet evident. Further work will be necessary to examine whether metastases are suppressed because 1α(OH)$D_3$ arrests the release of tumour cells from the primary neoplasm or because their secondary implantation and development are impaired.

**References**

ABE, E., MIYAURA, C., SAKAGAMI, H. & 5 others. (1981). Differentiation of mouse myeloid leukemia cells induced by 1α,25-dihydroxyvitamin $D_3$. *Proc. Natl Acad. Sci.*, **78**, 4990.

BRUMBAUGH, P.F. & HAUSSLER, M.R. (1975). Specific binding of 1α,25-dihydroxycholecalciferol to nuclear components of chick intestine. *J. Biol. Chem.*, **250**, 1588.

COLSTON, K., COLSTON, M.J. & FELDMAN, D. (1981). 1,25-dihydroxyvitamin $D_3$ and malignant melanoma: The presence of receptors and inhibition of cell growth in culture. *Endocrinology*, **108**, 1083.

FRAMPTON, R.J., SUVA, L.J., EISMAN, J.A. & 4 others. (1982). Presence of 1,25-dihydroxyvitamin $D_3$ receptors in established human cancer cell lines in culture. *Cancer Res.*, **42**, 1116.

FUKUSHIMA, M., SUZUKI, Y., TOHIRA, Y. & 5 others. (1975). Metabolism of 1α-hydroxyvitamin $D_3$ to 1α-25-dihydroxyvitamin $D_3$ in perfused rat liver. *Biochem. Biophys. Res. Commun.*, **66**, 632.

HONMA, Y., HOZUMI, M., ABE, E. & 6 others. (1983). 1α,25-dihydroxyvitamin $D_3$ and 1α-hydroxyvitamin $D_3$ prolong survival time of mice inoculated with myeloid leukemia cells. *Proc. Natl Acad. Sci.*, **80**, 201.

SATO, T., TAKUSAGAWA, K., ASOO, N. & KONNO, K. (1982). Antitumor effect of 1α-hydroxyvitamin $D_3$. *Tohoku J. Exp. Med.*, **138**, 445.

SATO, T. & SATO, H. (1983). Establishment of transplantable ascites hepatomas induced in ACI/N rats by N-diethylnitrosamine. *Sci. Rep. Res. Inst. Tohoku. Univ-C.*, **30**, 15.